# Disability Identity and Perceptions of Institutional Fairness and Climate in Academic Medicine

**DOI:** 10.1001/jamanetworkopen.2024.30367

**Published:** 2024-08-28

**Authors:** Jonathan Altamirano, Magali Fassiotto, Arghavan Salles, Ken Sutha, Yvonne Maldonado, Peter Poullos

**Affiliations:** 1 Department of Epidemiology and Population Health, Stanford University School of Medicine, Stanford, California; 2 Office of Faculty Development and Diversity, Stanford University School of Medicine, Stanford, California; 3 Department of Medicine, Stanford University School of Medicine, Stanford, California; 4 Department of Pediatrics, Stanford University School of Medicine, Stanford, California; 5 Department of Radiology, Stanford University School of Medicine, Stanford, California

## Abstract

This cross-sectional study examines whether students and employees with or without disabilities at a major academic medical center in the US feel respected and are treated equitably at their institution

## Introduction

Although the Americans with Disabilities Act (ADA) has been in effect for over 3 decades, challenges persist for professionals with disabilities, including lower employment rates than their peers,^1^ and those with disabilities who are employed experience underemployment, part-time or contingent employment, and lower mean salaries.^1,2^ These data suggest that people with disabilities likely experience workplaces differently from those who do not have disabilities.^3^ People with disabilities are also underrepresented in academic medical centers, as only 5% of faculty identify as having disabilities,^4^ compared with 14% of medical students,^5^ indicating a loss of trainees along the career path. To better understand the experiences of health care professionals with disabilities, we asked students and employees at a major US academic medical center whether they feel respected and are treated equitably at their institution.

## Methods

This cross-sectional study was conducted at Stanford Medicine from November 1 to December 5, 2020. Faculty, students or trainees, and staff were invited to participate in an anonymous online survey. Survey elements included disability identity (no disability, ADA-defined disability and self-identify as disabled, or ADA-defined disability but did not self-identify as disabled; eAppendix in Supplement 1), demographics (gender, race and ethnicity, and sexual orientation; see Table 1 for full list of demographic responses), and 5 climate statements regarding feelings toward the institution, rated on a 5-point Likert scale (Table 2). This study was deemed exempt and consent was waived by the Stanford University School of Medicine’s institutional review board because the data were deidentified and collected anonymously as part of a needs assessment. No identifying information was collected and surveys were answered completely anonymously via survey link distributed through listservs. This study followed the STROBE reporting guideline.

**Table 1.** Participant Demographic Characteristics

Characteristic	Participants, No. (%)	
	Total sample (N = 3311)	Included in analysis (n = 2912)
Gender		
Man	846 (25.5)	771 (26.5)
Woman	2317 (70.0)	2078 (71.4)
Other^a^	148 (4.5)	63 (2.2)
Sexual orientation		
Sexual minority^b^	384 (11.6)	342 (11.7)
Straight or heterosexual	2805 (84.7)	2567 (88.2)
Declined to state	122 (3.7)	3 (0.1)
Race and ethnicity		
Asian	746 (22.5)	653 (22.4)
URiM	686 (20.7)	600 (20.6)
American Indian or Alaska Native	8 (0.2)	0
Black or African American	155 (4.7)	145 (5.0)
Hispanic or Latine	406 (12.3)	376 (12.9)
Native Hawaiian or Pacific Islander	36 (1.1)	0
>2 URiM races and ethnicities	81 (2.4)	79 (2.7)
White	1680 (50.7)	1588 (54.5)
Other^c^	199 (6.0)	71 (2.4)
Disability status		
Self-identified as disabled	267 (8.1)	238 (8.2)
ADA-defined disability	625 (18.9)	577 (19.8)
No reported disability	2419 (73.0)	2097 (72.0)

Abbreviations: ADA, Americans with Disabilities Act; URiM, underrepresented in medicine.

^a^ Includes transgender, gender nonconforming, gender nonbinary, genderqueer, or other gender identity.

^b^ Includes lesbian, gay, bisexual, queer, or other.

^c^ The survey answer of “Other” did not specify races or ethnicities.

**Table 2.** Adjusted Odds Ratios for the Association Between Disability Identity and Agreement With Climate and Inclusion Statements^a^

Statement	Participants, No./total No. (%)		Adjusted odds ratio (95% CI)
	Neutral or disagreement	Agreement	
**“I feel connected to the vision, mission, and values of my institution” (n = 2714)**			
No reported disability	285/1969 (14.5)	1684/1969 (85.5)	1 [Reference]
ADA-defined disability	98/535 (18.3)	437/535 (81.7)	0.77 (0.59-0.99)
ADA-defined disability and self-identify as disabled	49/210 (23.3)	161/210 (76.7)	0.57 (0.40-0.82)
**“In my workplace or learning environment, I experience respect among individuals and groups with various cultural differences” (n = 2718)**			
No reported disability	199/1976 (10.1)	1777/1976 (89.9)	1 [Reference]
ADA-defined disability	82/532 (15.4)	450/532 (84.6)	0.62 (0.47-0.82)
ADA-defined disability and self-identify as disabled	40/210 (19.1)	170/210 (80.9)	0.52 (0.35-0.77)
**“In my institution, I am confident that my accomplishments are compensated or rewarded similarly to others who have achieved their goals” (n = 2258)**			
No reported disability	439/1664 (26.4)	1225/1664 (73.6)	1 [Reference]
ADA-defined disability	140/442 (31.7)	302/442 (68.3)	0.75 (0.60-0.95)
ADA-defined disability and self-identify as disabled	54/152 (35.5)	98/152 (64.5)	0.66 (0.46-0.95)
**“I trust my institution to be fair to all employees and students” (n = 2258)**			
No reported disability	439/1664 (26.4)	1225/1664 (73.6)	1 [Reference]
ADA-defined disability	140/442 (31.7)	302/442 (68.3)	0.83 (0.65-1.05)
ADA-defined disability and self-identify as disabled	54/152 (35.5)	98/152 (64.5)	0.62 (0.44-0.89)
**“If I raised a concern about discrimination, I am confident my institution would do what’s right” (n = 2376)**			
No reported disability	569/1764 (32.3)	1195/1764 (67.7)	1 [Reference]
ADA-defined disability	152/454 (33.5)	302/454 (66.5)	0.94 (0.75-1.18)
ADA-defined disability and self-identify as disabled	64/158 (40.5)	94/158 (59.5)	0.71 (0.50-0.99)

Abbreviation: ADA, Americans with Disabilities Act.

^a^ Five-point Likert scales were used for each question, ranging from strongly disagree to strongly agree. In preparation for logistic regression, the 5-point scale was collapsed into a binary variable: (1) strongly disagree, disagree, or neither agree nor disagree vs (2) agree or strongly agree. Each statement was run in its own model. All models were adjusted for participant gender (man, woman, and other gender identities), race and ethnicity (Asian, Black or African American, Hispanic or Latine, White, and ≥2 races), and sexual orientation (sexual minority and straight or heterosexual).

Statistical analysis was performed from January 9 to June 30, 2023. Associations between disability identity and agreement with each statement (“agree” and “strongly agree” combined) were evaluated using logistic regression models adjusted for participant demographics. Odds ratios (ORs) and 95% CIs are reported here. All tests were 2-sided, with *P* < .05 considered statistically significant. Analyses were performed in SAS, version 9.4.

## Results

Of 3311 participants discussed in a prior publication,^4^ 2912 completed at least 1 climate question and were included in this analysis (Table 1). Participants who self-identified as being disabled and had an ADA-defined disability showed significantly lower odds of agreeing with every climate statement compared with individuals with no reported disability (question 1: OR, 0.57 [95% CI, 0.40-0.82]; question 2: OR, 0.52 [95% CI, 0.35-0.77]; question 3: OR, 0.66 [95% CI, 0.46-0.95]; question 4: OR, 0.62 [95% CI, 0.44-0.89]; question 5: OR, 0.71 [95% CI, 0.50-0.99]) (Table 2). Participants with an ADA-defined disability who did not self-identify as disabled also had significantly lower odds of agreeing with the first 3 statements (question 1: OR, 0.77 [95% CI, 0.59-0.99]; question 2: OR, 0.62 [95% CI, 0.47-0.82]; question 3: OR, 0.75 [95% CI, 0.60-0.95].

## Discussion

To our knowledge, this is the first major study of disabilities and equity across a major health center in the US. Respondents who self-identified as having a disability and had an ADA-defined disability consistently showed lower odds of agreeing with positive institutional statements, in line with prior research.^6^ These findings highlight a significant gap between the inclusivity goals of institutions and how those with disabilities perceive and experience work at these institutions.

This study is limited by its cross-sectional design, and an accurate response rate cannot be calculated because the survey was distributed through anonymous listservs. These data serve as a call to action for health care institutions to evaluate their internal policies and to foster an environment that not only communicates values of equity and inclusion but ensures these values are felt and experienced by all members. Ensuring that all employees, regardless of disability status, gender, race and ethnicity, or any other identity, feel a genuine connection to their institution’s vision and mission is crucial for creating truly inclusive environments.

## Notes

Supplement 1. **eAppendix.** Item S1. Survey Question: Determination of Americans With Disabilities Act (ADA)–Defined Disability

Supplement 2.
Data Sharing Statement
