# Supplementary material for: Disability Identity and Perceptions of Institutional Fairness and Climate in Academic Medicine
Source: JAMA Netw Open. 2024 Aug 28;7(8):e2430367. doi: 10.1001/jamanetworkopen.2024.30367 (PMC11358856; doi:10.1001/jamanetworkopen.2024.30367)
Supplement: Supplement 1. — eAppendix. Item S1. Survey Question: Determination of Americans With Disabilities Act (ADA)–Defined Disability [file jamanetwopen-e2430367-s001.pdf]

Supplementary Online Content

Altamirano J, Fassiotto M, Salles A, Sutha K, Maldonado Y, Poullos P. Disability identity and perceptions of institutional fairness and climate in academic medicine. *JAMA Netw Open.* 2024;7(8):e2430367. doi:10.1001/jamanetworkopen.2024.30367

eAppendix. Item S1. Survey Question: Determination of Americans With Disabilities Act (ADA)–Defined Disability

This supplementary material has been provided by the authors to give readers additional information about their work.

© 2024 Altamirano J et al. *JAMA Network Open.*

eAppendix. Item S1. Survey Question: Determination of Americans With Disabilities Act (ADA)–Defined Disability

Definition of Disability - Americans with Disabilities Act (ADA) An individual with a disability is defined as someone who has "a physical or mental impairment that substantially limits one or more major life activities; has a record of such an impairment; or is regarded as having such an impairment."

The American with Disabilities Act regulations define "physical impairment" as any cosmetic disfigurement, or anatomical loss affecting one or more body systems, such as neurological, musculoskeletal, special sense organs, respiratory (including speech organs), cardiovascular, reproductive, digestive, genitourinary, immune, circulatory, hematologic, lymphatic, skin, and endocrine.

The regulations also cover any mental or psychological disorder, such as intellectual disability, organic brain syndrome, emotional or mental illness and specific learning disabilities. Although there is not an exhaustive list of disabilities under the American with Disabilities Act (ADA), the regulations identify medical conditions that would easily be considered a disability within the meaning of the law. Some conditions that would be considered a disability include but are not limited to the following:

Deafness or Other Hearing Loss	HIV infection
Blindness or Low Vision	Multiple Sclerosis
Diabetes	Muscular Dystrophy
Cancer	Major Depressive Disorder
Epilepsy	Bipolar Disorder
Intellectual Disability	Post-Traumatic Stress Disorder
Partial or Completely Missing Limbs	Obsessive-Compulsive Disorder
Autism	Schizophrenia
Attention Deficit Disorder (ADD)	Mobility Limitations
Attention Deficit Hyperactivity Disorder (ADHD)	Cerebral Palsy
Dyslexia	Speech and Language Disabilities
Other Learning Disabilities	Chronic Illness
Orthopedic or Rheumatologic Conditions	Autoimmune Disorders

By the above definition, do you have a medical condition that would qualify as a disability?

A. Yes, and I identify as a person with a disability or disabled
B. Yes, but I do not identify as a person with a disability or disabled
C. No, but I do identify as a person with a disability or disabled
D. No, I do not have a condition that could be considered a disability

© 2024 Altamirano J et al. *JAMA Network Open.*
