# Supplementary material for: Disability Identity and Perceptions of Institutional Fairness and Climate in Academic Medicine
Source: JAMA Netw Open. 2024 Aug 28;7(8):e2430367. doi: 10.1001/jamanetworkopen.2024.30367 (PMC11358856; doi:10.1001/jamanetworkopen.2024.30367)
Supplement: Supplement 2. — Data Sharing Statement [file jamanetwopen-e2430367-s002.pdf]

Data Sharing Statement

Altamirano. Disability Identity and Perceptions of Institutional Fairness and Climate in Academic Medicine. *JAMA Netw Open*. Published August 28, 2024.
doi:10.1001/jamanetworkopen.2024.30367

Data
Data available: No

Additional Information
Explanation for why data not available: Authors would be happy to do additional data queries for interested individuals. However, the authors feel uncomfortable making the data openly available as the data was collected under waiver of consent
